# Supplementary material for: Framework for identifying drug repurposing candidates from observational healthcare data
Source: JAMIA Open. 2020 Dec 31;3(4):536–44. doi: 10.1093/jamiaopen/ooaa048 (PMC7886555; doi:10.1093/jamiaopen/ooaa048)
Supplement: ooaa048_Supplementary_Data [file ooaa048_supplementary_data.docx]

# Supplemental material for “Framework for Identifying Drug Repurposing Candidates from Observational Healthcare Data”

## Causal Inference Methods Applied in the PD Case Study

### Estimating the Causal Effect of the Trial Drug

Our framework considers two types of biases that may hamper the estimation of causal effects. The first is confounding, caused by features affecting both the treatment assignment and the outcome. The second is informative censoring, caused by an association between incomplete follow-up and the outcome analyzed in the trial. In the PD case study, we applied two common approaches for estimating causal effects: (i) balancing weights; and (ii) outcome prediction. We considered a drug as *strongly* beneficial for PD if it is estimated to have significant beneficial effect by these two methods.

To describe the two approaches of estimating effects, we start by introducing some additional notations. Let $A_{i}$ indicate the treatment assignment for patient $i$, where $A_{i}=1$ denotes the trial drug (i.e., treatment cohort), and $A_{i}=0$ the alternative treatment (i.e., control cohort). Let $T_{i}^{\text{outcome}}$ be the time of the first outcome event (if the patient had no outcome event, we set $T_{i}^{\text{outcome}}=Inf$). Let $T_{i}^{C}$ be the censoring time, which is the end-of-treatment, and $T_{i}=min(T_{i}^{\text{outcome}}, T_{i}^{C})$. In the PD case study time was measured in months (30 days).

#### Balancing weights

In this approach we compute two types of patient weights: $w_{x_{i}}^{A}$, to correct for imbalances in selected confounders between treatment and control cohorts; and $w_{t,x_{i}}^{C}$, to correct for informative censoring at time $t$ within each cohort. We set $w_{t, x_{i}}=w_{x_{i}}^{A}*w_{t,x_{i}}^{C}$ and estimate the potential outcome probability for each treatment by the following weighted Kaplan-Meier estimator:

$$P^{a}\left( \text{outcome} \right)=\prod_{t=1}^{\text{follow-up length}} \left( 1- \frac{\sum_{\{i: A_{i}=\text{a\}}} w_{t, x_{i}}1[T_{i}^{\text{outcome}}=t]}{\sum_{\{i: A_{i}=\text{a\}}} w_{t, x_{i}}1[T_{i}\geq t]} \right)$$

where $1[condition]=1$ if the corresponding condition is correct and 0 otherwise.

The generation of balancing weights, $w_{x_{i}}^{A}$, was described above (see Section Causal Inference Library). We computed the weights $w_{t,x_{i}}^{C}$ to correspond to the inverse of each patient’s “tendency” to be uncensored at time $t$, by applying the method of inverse probability of censoring weights (IPCW) [1]. These weights were computed in each treatment cohort separately, to account for a potential independent censoring mechanism in each group. More formally, patient $i$ at time $t$ is assigned with a weight$w_{t,x_{i}}^{C}$defined as:

$$w_{t,x_{i}}^{C}= \frac{K_{0}(t)}{K_{x_{i}}(t)}$$

where

$$K_{0}\left( t \right)=\prod_{t^{'}=1}^{t-1} \left( 1- P\left[ T_{i}^{C}\geq t^{'} \right|T_{i}^{C}\geq t^{'}-1] \right)$$

$$K_{x_{i}}\left( t \right)=\prod_{t^{'}=1}^{t-1} \left( 1- P\left[ T_{i}^{C}\geq t^{'} \right|T_{i}^{C}\geq t^{'}-1, X=x_{i}] \right)$$

For computing $K_{0}\left( t \right)$ and $K_{x_{i}}\left( t \right)$ we estimated the conditional probabilities $P\left[ T_{i}^{C}\geq t \right|T_{i}^{C}\geq t-1]$ and $P\left[ T_{i}^{C}\geq t \right|T_{i}^{C}\geq t-1, X=x_{i}]$ respectively. Each of these conditional probabilities was estimated using a logistic regression model that predicts the risk for being censored in each time unit by pooling the observations from all time units. Note that patients with the outcome event, i.e. $T_{i}^{\text{outcome}}<Inf$, may contribute to the training of these models only till the time of the outcome event. In the PD case study, the predictors in the model for $P\left[ T_{i}^{C}\geq t \right|T_{i}^{C}\geq t-1]$ included the time-terms $t$ and $t^{2}$; in the model for $P\left[ T_{i}^{C}\geq t \right|T_{i}^{C}\geq t-1, X=x_{i}]$, the predictors included $t$, $t^{2}$ and the baseline features, $X$. Such models are known as pooled logistic regression models and were shown to approximate Cox proportional hazards models [2].

#### Outcome prediction

This approach trains a model to predict $P\left[ T_{i}^{\text{outcome}}=t | T_{i}^{\text{outcome}}\geq t, X=x_{i},A_{i}=a \right]$, and uses it to infer the expected outcome for each individual patient and treatment

$P\left[ \text{outcome} \right|X=x_{i}, A_{i}=a]=1-\prod_{t=1}^{T_{\text{follow-up}}} \left( 1-P\left[ T_{i}^{\text{outcome}}=t | T_{i}^{\text{outcome}}\geq t, X=x_{i}, A_{i}=a \right] \right)$

Let $n$ be the number of patients in the trial (i.e., in both treatment and control cohorts). The expected outcome is estimated by averaging the inferred individual outcomes on all patients in the trial:

$$P^{a}\left( \text{outcome} \right)=\frac{1}{n}\sum_{i} P\left[ \text{outcome} \right|X=x_{i}, A_{i}=a]$$

A common way to train a model for $P\left[ T_{i}^{\text{outcome}}=t | T_{i}^{\text{outcome}}\geq t, X=x_{i},A_{i}=a \right]$ is by pooling the observations from all time points (aka pooled model, [2]). In the PD case study, we used a pooled logistic regression with the following predictors: $X, A, \text{t},\text{ }\text{t}^{2}, A*X, A*\text{t},\text{ A*}\text{t}^{2}$. To correct for possible informative censoring, we trained the outcome prediction model after reweighting the pooled observations with IPCW.

### Significance Assessment

Given a certain estimator (e.g., for the causal effect), we assessed whether it was significantly larger or smaller than zero via parametric bootstrap. Specifically, we generated $N_{B}$ bootstrap samples of the patients in the trial by sampling with replacement. We used $N_{B}=100$ in Explorys and $N_{B}=200$ in MarketScan. We then estimated the standard error of the statistic (e.g., the treatment effect) by its standard deviation on the $N_{B}$ bootstrap samples. Using the estimated standard error, we computed a Z-score for the statistic from which a P-value was computed under the assumption of a normal distribution.

To account for multiple tests, we used the False Discovery Rate (FDR) method [3] to adjust all P-values associated with drugs effects (Step 12 in procedure 1). In the PD case study, these included the P-values computed by both causal effect estimation methods (i.e., balancing weights and outcome prediction) in both databases.


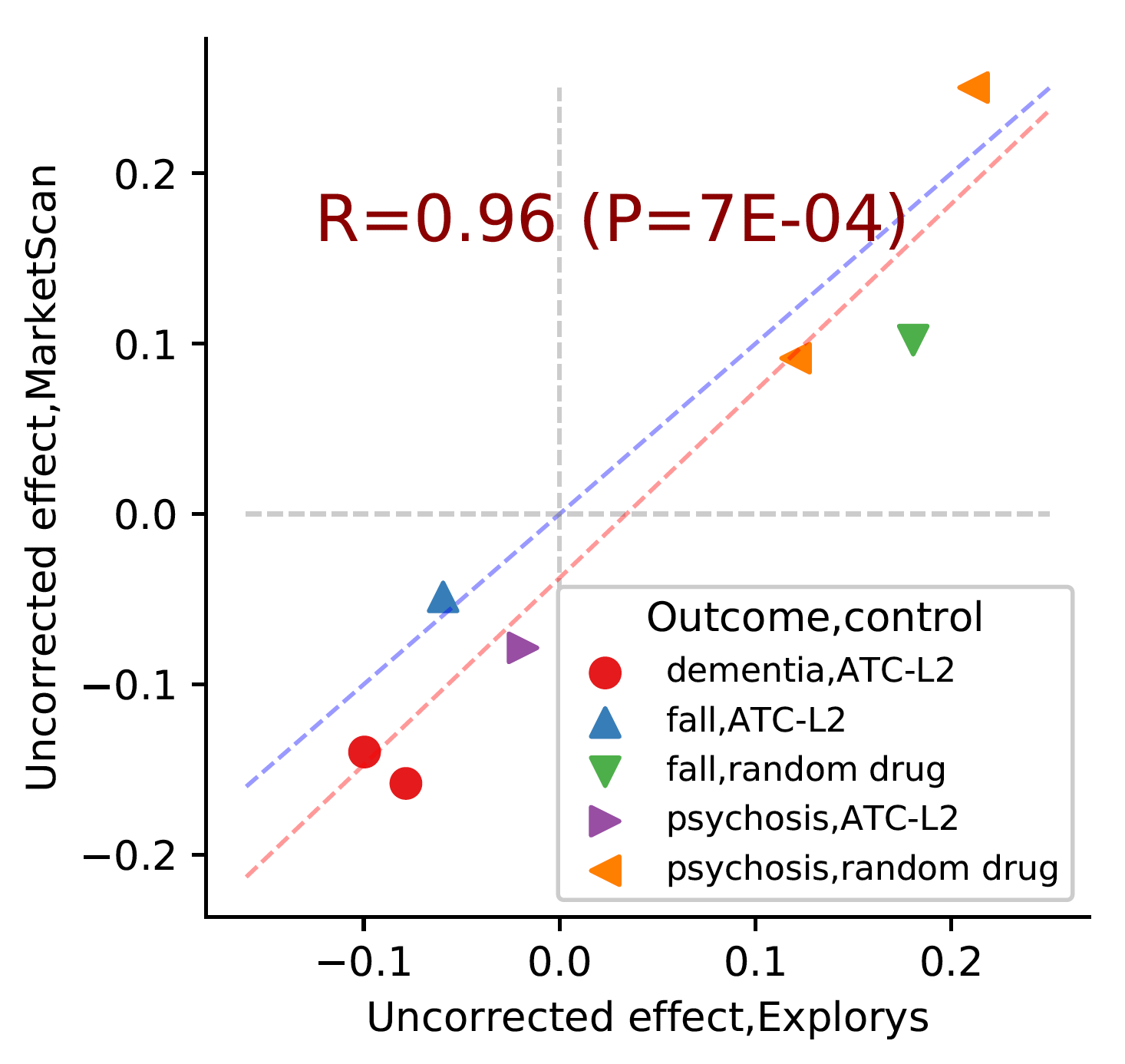


Supplemental Figure 1. The uncorrected effects corresponding to the estimated causal effects shown in Figure 4.

# References

1 Robins JM, Finkelstein DM. Correcting for noncompliance and dependent censoring in an AIDS Clinical Trial with inverse probability of censoring weighted (IPCW) log-rank tests. *Biometrics* 2000;**56**:779–88.

2 D’Agostino RB, Lee M-L, Belanger AJ, *et al.* Relation of pooled logistic regression to time dependent cox regression analysis: The framingham heart study. *Statistics in Medicine* 1990;**9**:1501–15. doi:10.1002/sim.4780091214

3 Benjamini Y, Hochberg Y. Controlling the False Discovery Rate: A Practical and Powerful Approach to Multiple Testing. *Journal of the Royal Statistical Society Series B (Methodological)* 1995;**57**:289–300. doi:10.2307/2346101
